# Optimal speed estimation in natural image movies predicts human performance

**DOI:** 10.1038/ncomms8900

**Published:** 2015-08-04

**Authors:** Johannes Burge, Wilson S. Geisler

**Affiliations:** 1 Department of Psychology, University of Pennsylvania, Philadelphia, Pennsylvania 19104, USA; 2 Center for Perceptual Systems, University of Texas at Austin, Austin, Texas 78712, USA

## Abstract

Accurate perception of motion depends critically on accurate estimation of retinal motion speed. Here we first analyse natural image movies to determine the optimal space-time receptive fields (RFs) for encoding local motion speed in a particular direction, given the constraints of the early visual system. Next, from the RF responses to natural stimuli, we determine the neural computations that are optimal for combining and decoding the responses into estimates of speed. The computations show how selective, invariant speed-tuned units might be constructed by the nervous system. Then, in a psychophysical experiment using matched stimuli, we show that human performance is nearly optimal. Indeed, a single efficiency parameter accurately predicts the detailed shapes of a large set of human psychometric functions. We conclude that many properties of speed-selective neurons and human speed discrimination performance are predicted by the optimal computations, and that natural stimulus variation affects optimal and human observers almost identically.

## Abstract (web summary)

Accurate motion perception depends on accurate estimation of retinal motion speed. Here, from natural image movies, the authors derive the optimal computational rules for estimating speed, and show that these computations predict both human speed discrimination performance and the tuning of speed-selective neurons.

## Graphical abstract

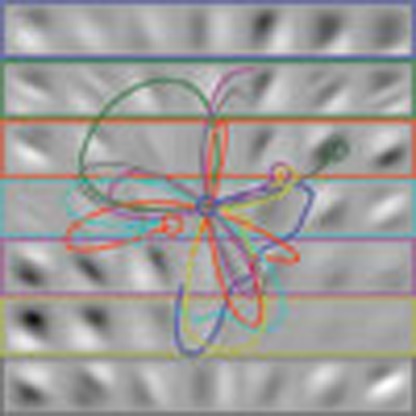

Accurately encoding the three-dimensional structure of the environment, and the motion of objects and the self through the environment requires accurately estimating local properties of the retinal images such as defocus blur, binocular disparity, motion direction and motion speed. Accurate estimation of these local properties is difficult because of the enormous complexity and variability of natural images. Thus, a major goal of early and mid-level visual processing must be to accurately estimate key retinal image properties despite the irrelevant variation in natural retinal images—a form of sensory-perceptual constancy. Here we consider the task of estimating the speed of local retinal image motion created by natural image movies. Accurately encoding motion signals is critical in sighted organisms for navigating the environment and reacting appropriately to predators and prey.

Explicit encoding of local retinal image motion begins early in the visual system^1^,^2^. In monkeys, and presumably in humans, many simple and complex cells in primary visual cortex (V1) are selective for the sign of motion direction^2^,^3^,^4^,^5^,^6^. Influential models have been proposed to account for this selectivity^4^,^7^,^8^,^9^,^10^. Simple-cell models are often defined by a linear space-time receptive field (RF)^8^,^9^,^10^ and complex-cell models by the sum of the squared responses from an appropriate pair of linear space-time RFs^9^. However, these standard models are poorly tuned for speed, especially when stimulated by natural images.

More sophisticated models are required to account for the greater speed-selectivity that is characteristic of some V1 complex cells and middle temporal (MT) neurons. Model neurons that are selective for both the speed and the angular direction of motion can be obtained by combining the outputs of appropriate V1 simple and complex cells^11^,^12^,^13^,^14^. In these models, however, the RF shapes are typically chosen for mathematical convenience, and the combination rules are based on intuitive computational principles. Neither the RFs nor combination rules are based on measurements of natural signals. Thus, although these models account for many of the response properties of cells in V1 and MT, it is not known whether neurons having these properties provide the best substrate for motion estimation with natural stimuli. It may be that the response properties of the cells in V1 and MT underlying motion estimation are better described by mechanisms optimized for natural signals.

How should one determine the best linear (simple cell) RF properties? One popular approach has been to examine which the linear RFs provide an efficient sparse encoding of natural image movies^15^,^16^. This approach hypothesizes that motion selectivity (and all the other kinds of selectivity) in V1 neurons result from a coding scheme that optimizes a single general cost function that enforces sparseness and completeness, thereby encoding a faithful and energy efficient representation of the retinal image. Interestingly, when applied to natural image movies, this coding scheme produces a set of linear RFs that has some of the motion selective properties of V1 neurons (but see the study by Ringach^17^). However, given this general cost function, there is no reason to expect that such RFs would be particularly well-suited for motion estimation or any other specific task. Also, because sparse coding of moving images does not address which of the set of RF responses should be combined or how, it does not, by itself, say how motion should be estimated from natural image movies.

Our view is that task-focused studies of information processing may help us understand V1 processing better than sparse coding. This view is informed by the hypothesis that V1 neurons were shaped through evolution and development to support the specific tasks the organism must perform to survive and reproduce. Thus, V1 may be comprised of a mixture of different sub-populations, each supporting one—or a small number of—sensory-perceptual task. This is an empirical matter, but some evidence supports it. For example, the subset of V1 complex cells that project to motion area MT are more selective for the sign of motion direction^18^, and for motion speed^14^ than the average complex cell (or simple cell) in V1.

The approach advanced here (Fig. 1) is informed by the second hypothesis. Specifically, we developed an ideal observer for speed estimation by analysing movies derived from natural images. The first step of the analysis determines the set of linear space-time RFs that encode the retinal image information most useful for estimating speed in a given direction. The retinal image information incorporates the front-end effects of the optics, photoreceptor spacing, photoreceptor temporal integration and gain control, which we take directly from the physiological literature. Given these optimal linear RFs, the second step of the analysis determines the rules for optimally combining the responses of these optimal RFs (which are not speed-selective) to obtain a population of speed-selective units with arbitrary preferred speeds. Finally, standard population decoding of these speed-selective units yields precise, unbiased, optimal speed estimates (given the front-end constraints and optimal linear RFs).

Variation in stimulus structure is an important factor limiting the performance of the ideal observers. To see whether ideal and human performance is limited in the same way by this variation, we pitted ideal and human observers against each other in matched speed discrimination tasks. Both types of observers were shown a large random set of natural image movies; a given movie was never shown twice. Human performance paralleled ideal performance, although ideal performance was somewhat more precise. More remarkably, a single free parameter, which can represent the effects of a multiplicative internal noise (or some other form of computational inefficiency), yielded a close quantitative match between human and ideal observer performance.

Our computational analysis and behavioural data show that a task-specific analysis of natural signals predicts many properties of speed-selective neurons in V1 and MT, and many details of human speed discrimination performance. Critically, the optimal speed processing rules of the ideal observer are not arbitrarily chosen to match the properties of neurophysiological processing, nor are they fit to match behavioural performances. Rather, they are dictated by the task-relevant statistical properties of complex natural stimuli.

## Results

### Natural motion stimuli

Our ideal observer analysis and psychophysical experiment requires sets of movies for which the speeds are precisely known. We generated several movie sets from calibrated photographs of natural scenes^19^,^20^. Movie durations were 256 ms, a typical fixation duration. Movie speeds ranged from −8 to 8 deg/s, an ecologically relevant range. Figure 2a shows the retinal speeds produced by a natural scene as an observer walks past at a brisk pace (3.0 mph), while fixating a point on the ground; the distance to the nearest point in the scene is 7.0 m.

Movies were created by texture-mapping randomly selected patches of calibrated natural image onto planar surfaces, and then moving the surfaces behind a stationary 1.0 deg aperture. Slanted surfaces create non-rigid and frontoparallel surfaces create rigid retinal image motion. We performed our ideal observer analysis on movies with a distribution of slanted surfaces (non-rigid motion) similar to those in natural scenes^20^,^21^, and on frontoparallel surfaces (rigid motion) only. There was only a minor difference between the two (Supplementary Fig. 1). Hence, this method for simulating natural movies should be representative of many (but not all) cases that occur under natural conditions^21^. Future work (see Discussion) must address how occlusions (discontinuous motion) affect optimal sensor design^22^.

Figure 2b shows frames from several movies. These examples illustrate the substantial variation in stimulus structure that occurs across natural movies. We will see that this variation is a critically important factor limiting the performance of the ideal observer, and that ideal and human performance is limited in the same way by this variation.

The aim of our study was to determine an ideal observer for estimating speed in a given direction (‘component-motion speed')^15^,^16^,^23^,^24^,^25^, and to test whether human performance with natural image movies is predicted from this ideal observer. Movies were thus restricted to one-dimension of space by vertically averaging each frame of the movie (Fig. 2c). Our movies can thus be thought of as more natural versions of typical component-motion stimuli (sinewave gratings) or less natural (vertically averaged) versions of movies taken of the natural environment. The vertically averaged movies can be represented in standard space-time plots^9^. Note that vertically oriented RFs respond identically to original and vertically averaged movies. Conceptually, then, the present analysis is equivalent to determining how to optimally estimate speed within and from a single orientation column in V1 (Fig. 2c). Estimates of both speed and angular direction could be obtained by combining component-speed estimates from different orientation columns (see Discussion). As speed increases from zero, the space-time plot for a given patch becomes more tilted (Fig. 2b,d). The structure of the space-time plot varies greatly even across movies with the same speed (Fig. 2d).

### Optimal speed estimation

The first step in deriving the ideal observer for speed estimation is to discover the population of linear RFs that are optimal for speed estimation. We use a Bayesian method for dimensionality reduction called accuracy maximization analysis (AMA)^26^. AMA finds the linear RFs that on average across stimuli extract the information most useful for estimating the stimulus property of interest (in the present case, motion speed). For any fixed number of RFs and a given training set of stimuli, AMA returns the set of RFs that support maximal accuracy in the specific task (see Methods; code available: http://burgelab.psych.upenn.edu).

Before applying AMA, we make the information available to the ideal observer comparable to that available in human visual system. Specifically, we incorporate the eye's optics, and the wavelength sensitivity, spatial sampling and temporal impulse–response function of the photoreceptors^27^,^28^, together with luminance normalization (light adaptation) so that the responses are a spatiotemporal contrast signal **c**. We also add a small baseline amount of white spatiotemporal Gaussian noise **n** to the photoreceptor responses. This noise must be included to prevent AMA from learning to use information that is so weak as to be undetectable to the human visual system. We pick the level of noise *σ* to be consistent with the maximum human contrast sensitivity, although the specific value has relatively little effect on the results. Finally, we scale the noisy input signals to a vector magnitude of 1.0. This operation is consistent with the contrast normalization (contrast gain control) seen in the early visual system^10^,^29^,^30^ (Supplementary Note 1). Thus, this is also an appropriate biological constraint.

Given the above constraints, the response of an arbitrary linear RF is given by


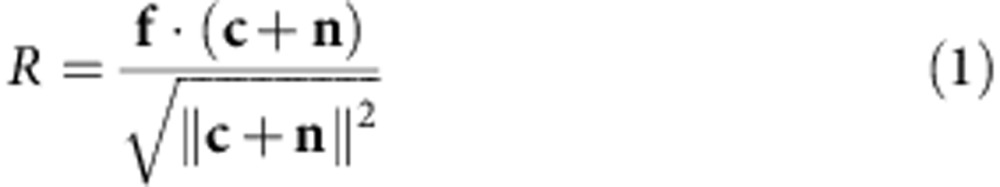


where **f** is a vector of the spatiotemporal pattern of weights (the RF), **c** is the input spatiotemporal contrast signal (vector), **n**∼*N*(0,*σ*^2^**I**) is a vector of i.i.d. Gaussian noise with s.d. *σ*, and ||**c**+**n**|| is the L2 norm (magnitude) of the noisy contrast vector. AMA finds the RF population **f**=(**f**_1_,⋯,**f**_*q*_) of size *q* that maximizes estimation accuracy. (Interestingly, the expectation of equation (1) closely approximates the standard equation in the literature for mean neural response as a function of image contrast; see Supplementary Note 1, Supplementary Fig. 1).

The specific population of eight spatiotemporal RFs that optimally encodes information for speed estimation is shown in Fig. 3a (Supplementary Fig. 2). Increasing the number of RFs beyond eight provides relatively little additional information. Some of these RFs are selective for the sign of motion direction (like many V1 simple cells, they are oriented in space time). Perhaps more surprisingly, some of the RFs are not selective for the sign of motion direction (also like some V1 simple cells). None are strongly selective for speed.

To illustrate their lack of speed-selectivity, the speed-tuning curves of each RF are plotted in Fig. 3b. Each tuning curve was obtained by computing the mean of the squared response across all natural movies at each speed in the training set (similar results are obtained from half-squaring). Response variability is large compared with the change in mean response. This response variability is not due to intrinsic neural noise (which is set to zero for this plot). Rather, it is due to task-irrelevant variation in the natural signals. That is, the space-time RFs are not response invariant to stimulus dimensions other than speed. Units with these space-time RFs individually provide poor information about speed in natural viewing. The same is presumably true for their neurophysiological analogues. The lack of speed-selectivity, and the lack of invariance to nuisance stimulus properties, occurs because (i) natural image movies contain a range of spatial frequencies, (ii) each space-time RF is sensitive only to a narrow band of frequencies, and (iii) the responses of each RF are significantly modulated both by spatial-frequency content and by speed. Although each individual unit provides relatively poor information about speed, as a population, the RFs optimally extract the information in the movies for speed estimation.

Selectivity for speed and invariance for ‘nuisance' stimulus properties can be achieved by combining the responses from the population of RFs. To determine how to optimally combine responses, we examine the population response to a large training set of natural image movies, conditioned on each speed. Figure 3c shows the conditional response distributions of RFs **f**_1_ and **f**_2_ to thousands of movies in the training set, colour-coded by speed. Each symbol is the joint response to one movie. Clearly, responses cluster as a function of speed. The covariance of the conditional responses (Fig. 3c) carries almost all the information about speed. The mean responses carry little information; the means are all centered near 0.0. Thus, the joint (for example, pairs of) RF responses carry nearly all the information about speed, while the responses of individual RFs carry very little.

The population responses evoked by natural stimuli specify the optimal (non-linear) combination rules. In the present case (speed estimation), the joint conditional response distributions (the distribution of population responses conditioned on speed) are approximately Gaussian:


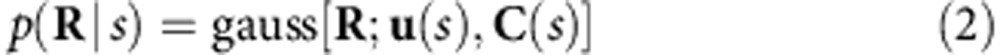


where **R** is the vector of eight linear RF responses given speed *s*. The vector of mean responses **u**(*s*) and the covariance matrix of responses **C**(*s*) are both functions of the speed. Given that the conditional response distributions are Gaussian, the distributions for each speed *s*_*k*_ are fully specified by the mean and covariance of the RF responses (equation (2)). Note that the conditional response distributions are Gaussian in part because of contrast normalization^21^,^31^; without normalization the distributions tend to be more kurtotic than Gaussian distributions^21^,^31^,^32^.

**Figure 1:**
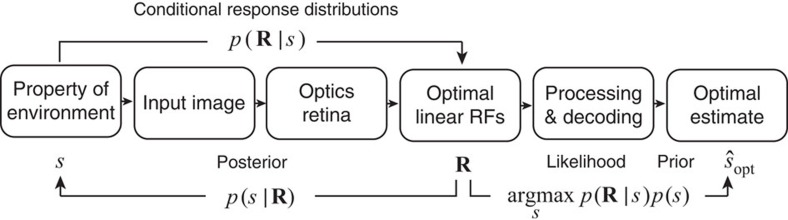
Ideal observer analysis. The task is to obtain the most accurate estimate of some property of the environment, in this case speed in a given direction (component speed). The first step is to find the set of linear receptive fields (RFs) that are optimal for speed estimation given natural image movies and known properties of the eye's optics and retina. These optimal RFs provide a set of responses **R** to each input image (note that a given speed can produce many different input images). The second step is to determine how the responses **R** should be combined to obtain the most accurate estimate of speed, which we take to be the maximum *a posteriori* (MAP) estimate (that is, the speed at which the product of the likelihood and the prior is at maximum).

The goal of the ideal observer is to pick the most probable speed given the observed population response; that is, the speed for which *p*(*s*|**R**) is greatest. This is the so-called maximum *a posteriori* decision rule. Applying Bayes' rule shows that the maximum *a posteriori* rule is equivalent to picking the speed for which *p*(**R**|*s*)*p*(*s*) is greatest (Fig. 1), where *p*(**R**|*s*) is the likelihood (L) (equation (2)), and *p*(*s*) is the prior probability distribution over speed. (Note that *p*(**R**|*s*) is the conditional response distribution when it is regarded as a function of **R** (equation (2)), and it is the likelihood function when regarded as a function of *s*). In the present experiment, the training and test movie sets had equal numbers of movies at each speed, and the human observers in the subsequent experiment (see below) knew that the speeds occur with equal probability. Hence, we assume a uniform prior. In this case, the ideal is to pick the speed for which the response vector has the greatest likelihood. Thus, the ideal observer is completely specified once the conditional response distributions are determined.

**Figure 2:**
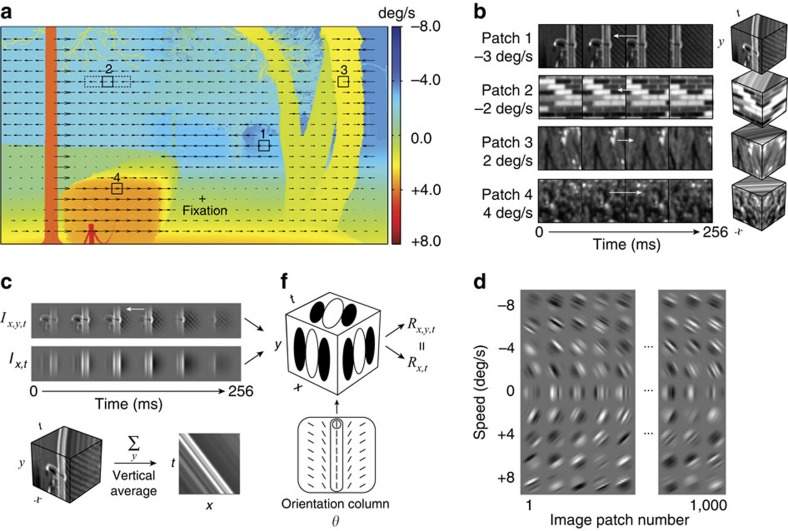
Naturalistic motion stimuli. (**a**) Speed of retinal image motion in a natural scene for an observer walking briskly to the left at 3.0 mph while fixating the marked point on the ground. A static range image was captured via laser time-of-flight. Distances to the objects in the scene range from 7 to 150 m. Four randomly selected image patches were the starting frame for each of the four movies (solid squares, the starting frame for each movie). (**b**) Movie image sequences and motion cubes from the image patches in **a**. Motion cubes show a three-dimensional (3D) space-time (*I*_*x,y,t*_) representation of movie. Different speeds (and different motion directions) correspond to different orientations in space-time (top surface of each cube). (**c**) Stimulus generation for computational analysis and behavioural experiment. Movies were vertically averaged and windowed to create a 2D space-time (*I*_*x,t*_) ‘component-motion' movie. Ideal and human observers were presented 2D movies. Vertically oriented space-time receptive fields, like those within an cortical orientation column, give identical responses (*ρ*=0.989; *y*=1.01*x*−0.02) to 2D and 3D movies. (**d**) Random samples from a large set of naturalistic space-time image movies that span a range of speeds (−8 to 8 deg/s). Variation within rows is due to textural variation in the movie (‘nuisance stimulus variation'). Variation across the rows is due to variation in speed.

**Figure 3:**
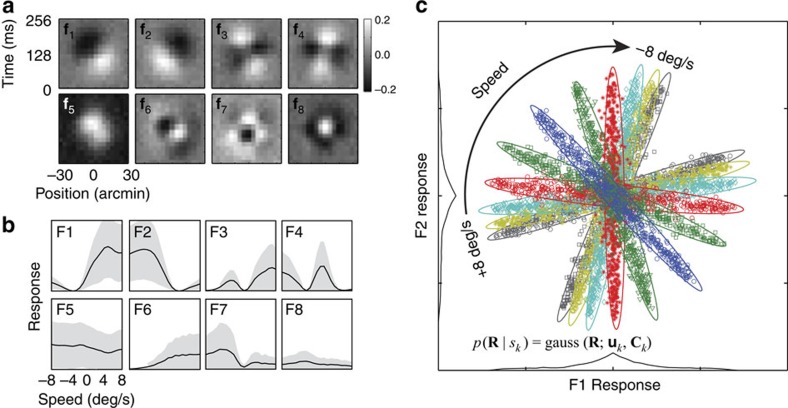
Optimal space-time receptive fields for speed estimation with naturalistic image movies. (**a**) Space-time receptive fields (RFs) that extract the most useful retinal image information for estimating speed. (**b**) Speed-tuning curves of the RFs in **a**. They are direction selective, but are not speed-tuned and they do not exhibit invariance. The grey area indicates response variability (±1 s.d.) due to irrelevant image features in natural images, not neural noise. (**c**) Conditional response distributions, *p*(**R** | *s*_*k*_), from the first two receptive fields. Different colours and symbols indicate different speeds. The information about speed is primarily contained in the covariance of the joint RF responses. As speed increases from zero, the distributions overlap more, suggesting that speed discrimination thresholds will increase with speed, similar to Weber's law for speed discrimination.

How might the optimal computations be implemented in neural circuits? The response distributions are approximately Gaussian (Fig. 3c); hence, the exponent of each Gaussian is a quadratic function of the RF responses. Therefore, a neural circuit that computes a weighted sum of squared RF responses and then passes the sum through an accelerating nonlinearity (Fig. 3a) could create a unit with responses that are proportional to the likelihood of the joint RF response (Fig. 2c) for a particular speed: 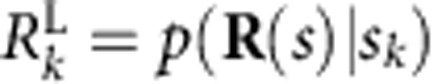 Appropriate construction of each of these L neurons depends crucially on determining the appropriate weights. The specific set of weights for each L neuron (which each has its own preferred speed) is specified by the covariance matrix **C**(*s*_*k*_) for that speed^21^ (see equation (2), Supplementary Note 2). Weighted summation and squaring are common operations in cortex. The final accelerating nonlinearity could be approximated by the non-linear relationship between membrane potential and spike rate. Thus, in principle, a population of speed-tuned neurons, in which each neuron has its own preferred speed *s*_*k*_, could be implemented with common neural computations.

The responses of many neurons in cortex are well-characterized by response models having multiple non-linear subunits (for example, linear RFs followed by a squaring nonlinearity) whose outputs are then combined linearly and passed through a final accelerating output nonlinearity^33^,^34^,^35^. Our analysis provides a normative prescription for determining the subunit RF structure, nonlinearities and weights that speed-selective neurons should have. Our approach thus has the potential to link methods for systems identification with normative principles for the design of circuits subserving particular tasks.

**Figure 4:**
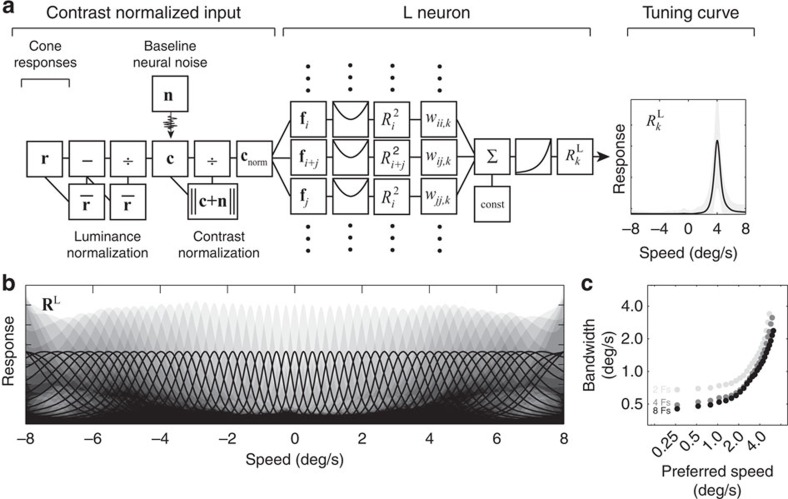
Likelihood neurons for speed estimation. (**a**) The response of each speed-tuned neuron, which represents the likelihood of its particular preferred speed, is obtained by appropriate weighted combination of the squared responses of the receptive fields (Fig. 3a). The weights are specified by the receptive field response distributions, conditioned on each speed (see Fig. 3c, Supplementary Note 2)). The speed-tuning curve of each likelihood neuron is much more selective for speed than the space-time receptive fields. (**b**) Likelihood neurons exhibit log-Gaussian speed-tuning curves, and are largely invariant to irrelevant features in the retinal images (grey area). Speed-tuning curve widths (speed bandwidths) increase systematically with preferred speed. These tuning curves are not cartoons. Rather, they are constructed directly from receptive field responses (see Fig. 3) to natural movies. (**c**) Preferred speed versus speed bandwidth increases with preferred speed. Tuning curve widths decrease systematically with an increase in the number of receptive fields (subunits) that are combined to produce the L neuron responses.

Speed-tuning curves for a population of L neurons spanning the whole range of speeds are shown in Fig. 4b. All tuning curves are unimodal, relatively invariant to the variable spatial structure of natural signals (Fig. 2d), and well-approximated by a log-Gaussian shape (except near zero). The bandwidth of speed-tuning curves increases with the absolute value of the neuron's preferred speed and decreases with increasing numbers of contributing space-time RFs (that is, subunits; Fig. 4c).

To determine the optimal speed estimate for a given movie from a population of speed-selective L neurons, we compute the response of each L neuron to the movie, interpolate the distribution of responses and read off the peak. The location of the peak is the optimal speed estimate. This decision rule is equivalent to reading off the peak of the posterior probability distribution under the assumption of a flat prior (that is, finding the max of equation (2) across speed). There already exist plausible neural computations for reading off the peak response of a neural population^36^.

**Figure 5:**
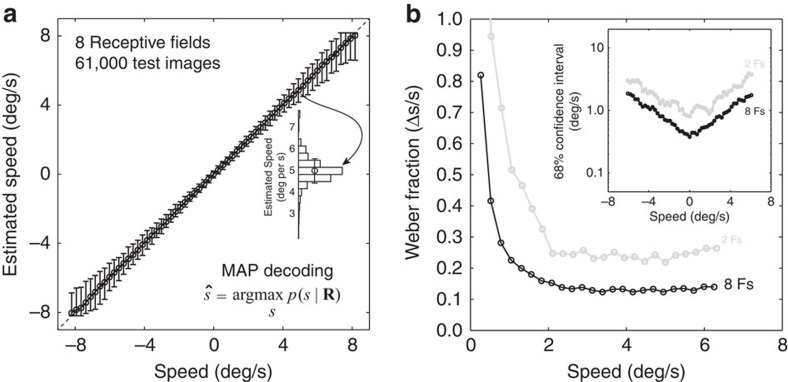
Optimal speed estimation and discrimination with test natural image movies. (**a**) Speed estimates as a function of speed. Error bars mark 68% confidence intervals (across natural stimuli having the same speed). The histogram shows the distribution of speed estimates for movies moving at 5 deg/s. (**b**) Weber fractions (Δ*s*/*s*) from the ideal observer using all eight (black) or only the first two (green) receptive fields (see Fig. 3a). Inset shows 68% confidence intervals on the estimates (∼2Δ*s*). Confidence intervals (∼ thresholds) increase exponentially with speed.

Speed estimation accuracy from a large collection of test natural movies (61,000 test patches: 1,000 natural inputs × 61 speeds) is shown in Fig. 5a. (None of the test patches were in the training set, and only one-third of the test speeds were in the training set). Speed estimates are unbiased over a wide range and error bars are quite small.

The ideal observer for speed estimation with natural stimuli has a pattern of speed discrimination thresholds (Fig. 5b) that is similar to the pattern characteristic of human performance with simple laboratory stimuli^18^,^25^,^37^,^38^. For both humans and ideal, as retinal speed increases the Weber fraction for speed discrimination decreases rapidly to an approximately constant value (generalized Weber's law).

### Comparison of human and ideal speed discrimination

The similarity between ideal observer performance (with natural stimuli) and human performance (with artificial stimuli) raises the following question: To what extent does ideal observer performance predict human performance with natural stimuli? To make the comparison precise, we measured human speed discrimination performance with a large random sample of the stimuli (7,000 movies per human observer) that were used to evaluate the ideal observer.

**Figure 6:**
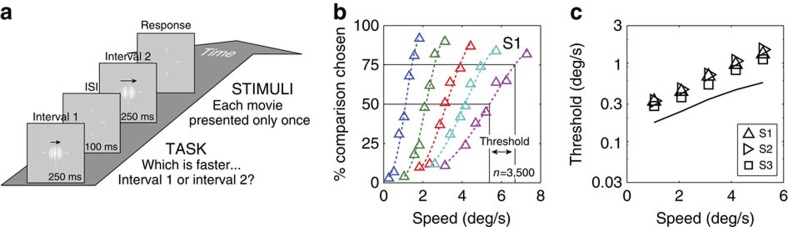
Measuring human speed estimation and discrimination performance. (**a**) Psychophysical task. (**b**) Psychometric functions for one observer at five standard speeds. The dashed curves are the cumulative normal distributions, fit via maximum likelihood estimation. (**c**) Speed discrimination thresholds (for *d*′=1.36) for the ideal (curve) and three human observers (symbols).

Speed discrimination psychometric functions were measured in a two-interval, two-alternative forced choice experiment (Fig. 6a). In each block of trials, the speed of one of the 256-ms movies was fixed (the standard), while the other movie (the comparison) had one of seven possible speeds. The spatiotemporal structure of both movies on every trial was always different; that is, throughout the entire experiment, the observer never saw the same movie twice (see Methods for details).

The psychometric functions of one human observer, measured at five standard speeds, are shown in Fig. 6b. The function slopes become shallower as the standard speed increases, indicating that speed discrimination becomes more difficult as speed increases. Thresholds for all three observers are similar at all standard speeds (symbols in Fig. 6c). Threshold is defined to be the difference between standard and comparison speeds that produces responses at 75% correct (*d*′=1.36). Human thresholds closely parallel those of the ideal (Fig. 6c, solid curve), although the humans are somewhat less sensitive (by a scale factor of 0.50–0.64). Both human and ideal thresholds increase exponentially with speed (that is, straight line on log-linear plot, which is close to the generalized Weber's law over this range of speeds—see Fig. 5b). This psychophysical law of speed discrimination has been observed repeatedly with artificial stimuli^25^,^37^,^38^. The present result shows (i) that the law holds with naturalistic stimuli and (ii) that the law follows from first principles (that is, a Bayesian ideal analysis of natural signals).

**Figure 7:**
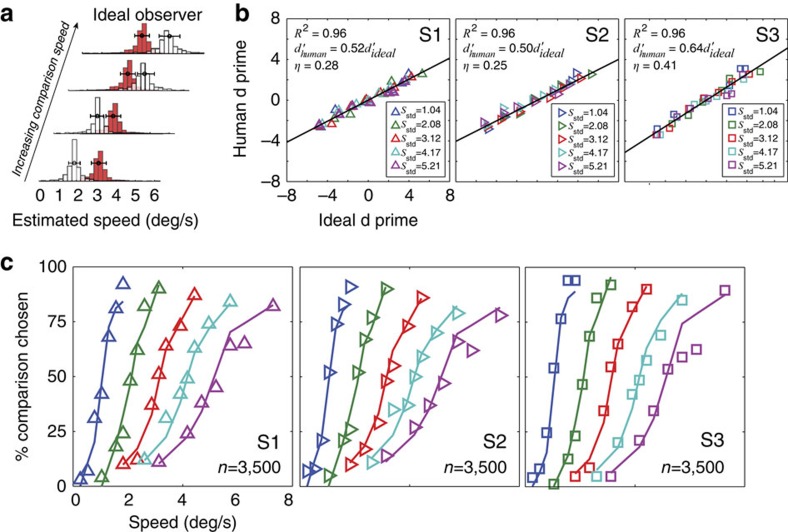
Human and ideal observer speed discrimination performance. (**a**) Estimate histograms (*c.f.*, Fig. 5a) from the ideal observer as a function of comparison speed. Ideal observer performance can be calculated directly from the estimate histograms by placing a criterion that will maximize percent correct. (**b**) Correlation of human and ideal observer performance for three human observers. Raw psychometric data (for example, Fig. 6b) was converted to sensitivity (*d*′) via the standard equation from signal detection theory: PC=Φ(*d*′/2). The efficiency *η* of each human observer corresponds to the squared slope of the best-fit line. (**c**) Human speed discrimination data with naturalistic image movies (symbols). The degraded ideal observer (coloured lines) accounts for each human's data across all conditions with a single free parameter.

Threshold measures of performance provide a useful summary of performance across multiple conditions, but they reduce each psychometric function to a single number. The raw psychometric data itself can be used to make richer, more detailed comparisons of human and ideal observers. First, we determine the proportion of times the ideal would choose the comparison stimulus (Fig. 7a). Next, for the both the human and ideal observers we convert proportion comparison chosen to signal-to-noise ratio (*d*′) using the standard formula *d*′=2Φ^−1^(PC), where PC is the percent comparison chosen^39^. (Negative *d*′ values correspond to conditions in which the observer chooses the comparison as faster less than 50% of the time. Discriminability increases with the magnitude of *d*′, independent of the sign.) Human sensitivity is highly correlated with ideal sensitivity across all standard and comparison speeds (Fig. 7b). Indeed, a single free parameter (that is, the slope of the best fit line in Fig. 7b) captures >95% of the variance in the data for each observer. This correspondence is remarkable given that the ideal observer, though constrained by front-end properties of the human visual system, was not designed to match human performance. The efficiency^40^ 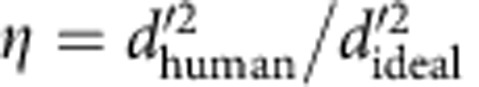 of each human observer, corresponds to the squared slope of the best-fitting line in each panel of Fig. 7b. Thus, a one-parameter model of human speed discrimination can be constructed by degrading the ideal observer to match human performance by some fixed computational inefficiency.

The predictions of the degraded ideal observer are shown in Fig. 7c. Fits were obtained by degrading ideal performance across all conditions by the efficiency of each observer: 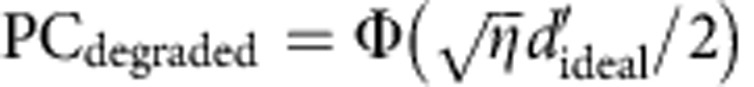. The detailed patterns in each human's raw psychometric data are nicely predicted with a single free parameter.

An analysis of trials in which standard and comparison speeds are identical shows that the degraded ideal predicts its own trial-by-trial responses at above chance (∼58%). This performance is rather low, but it is as expected given the efficiency, assuming it is due to internal noise. Human trial-by-trial responses predict degraded ideal responses (with standard and comparison identical) less well, but still significantly better than chance (∼53%).

**Figure 8:**
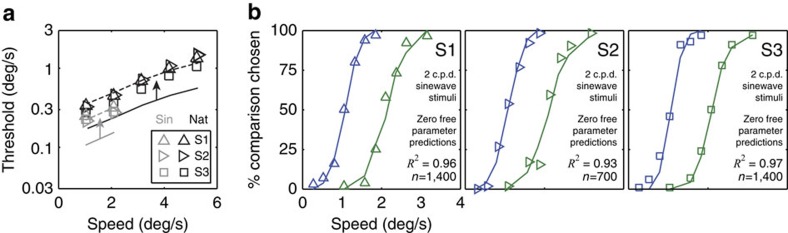
Human and ideal observer speed discrimination performance improves similarly when probed with drifting sinewaves. (**a**) Thresholds for discriminating speed with 2 c.p.d. sinewave stimuli (grey) versus natural stimuli (black). Human thresholds (symbols) and ideal thresholds (solid curves) decrease by approximately the same proportion (0.7 versus 0.63). Degrading ideal performance with both stimulus types by the same amount (arrows) produces a good fit of human performance (dashed curves). (**b**) The human psychometric data with sinewave stimuli (symbols) is predicted by the degraded ideal observer (solid curves). The efficiency parameter for the degraded ideal was obtained from the human data with natural stimuli (values of the efficiency parameter are given in Fig. 7b). The 30% decrease in human threshold with sinewave stimuli is quantitatively predicted with zero free parameters.

To further test the generality of the ideal observer and its predictions, we presented the ideal and human observers with classic artificial stimuli: drifting 2 c.p.d. sinewaves. With these sinewave stimuli, ideal (and degraded ideal) speed discrimination thresholds decrease by ∼30%. Human speed discrimination thresholds decrease by almost exactly the same amount (Fig. 8a). This result makes sense. Sinewave stimuli introduce less external variability than natural stimuli; hence, the lower thresholds. The degraded ideal observer, 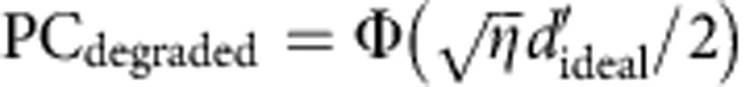, also accounts well for the detailed psychometric data. The symbols in Fig. 8b are the human data, and the solid curves are the predictions of the degraded ideal. The efficiency parameter for the degraded ideal was determined from the psychometric data with natural stimuli (Fig. 7b,c). The predictions in Fig. 8b were therefore obtained with zero free parameters.

## Discussion

An ideal observer for component-speed estimation was derived from an analysis of naturalistic image movies, given the constraints of the early visual system (optics, receptors, noise). We described the ideal computations, and showed how basic neural operations (for example, linear filtering, exponentiation, normalization) could produce neural populations that exhibit selectivity to speed and invariance to nuissance stimulus properties.

Then, we measured human and ideal performance in a speed discrimination task with the same set of natural image movies. Human discrimination thresholds paralleled but were somewhat higher than ideal thresholds across the range of tested speeds. More remarkably, the detailed shapes of the human psychometric functions were predicted by degrading the ideal observer's performance with a single free parameter (efficiency) across all conditions. After estimating human efficiency with the natural image movies, we tested human performance with drifting sinewaves. The degraded ideal predicts a substantial improvement in human thresholds (30%) with zero additional free parameters. The human data quantitatively conforms to these predictions.

Unlike other models of speed discrimination, the ideal observer was not constructed to match known facts about human speed discrimination performance. Rather, the behavioural predictions follow from a principled Bayesian analysis of naturalistic movies, given the constraints imposed by the visual system's front end. (We emphasize that the predictions depend on both the statistical structure of the stimuli and the front-end properties included in the analysis). Thus, despite some valid concerns that have been expressed in the literature^33^, our results suggest that the variation in natural signals can be used both to build principled models of visual computations and to effectively probe performance. Indeed, we find that some well-established properties of neurons involved in speed estimation and a fundamental law of speed discrimination performance (exponential law or approximate generalized Weber's law) follow directly from optimal computations on naturalistic movies.

Similar computations were recently found to be optimal for binocular disparity estimation with natural signals^21^. Disparity and motion estimation are fundamentally different tasks in early- and mid-level vision, each with its own extensive neurophysiological and psychophysical literatures. The success of the same approach in these two distinct domains of visual neuroscience may constitute an important scientific result in its own right. Specifically, it suggests the possibility that the computations in Fig. 4a together with near-optimal task-relevant linear RFs, may be a class of functional neural computation that the brain uses in other visual tasks and in other sensory modalities (a form of ‘canonical' neural computation^41^).

It has long been recognized that a goal of perceptual processing may be to transform sensory signals into a representation where specific dimensions of information are made more explicit^42^. For example, neurons in primary V1 make more explicit the orientation information implicitly contained in the retinal outputs. In the context of object recognition, a version of this hypothesis is the ‘untangling' hypothesis, which states that as visual processing proceeds, the neural representation of a variable of interest is transformed from a non-linearly separable to a linearly separable representation^43^. The present approach would seem to constitute a quantitative example of such an ‘untangling' process—it specifies the optimal rules for taking the ‘tangled' (linearly inseparable) representation of speed in the simple-cell-like RFs (Fig. 3c) and transforming it into an ‘untangled' (linearly separable) representation of speed in the speed-tuned neurons (Fig. 4). In other words, the population of speed-tuned neurons explicitly represents speed.

### Comparison with V1 and MT neurons

The first-level linear units that are somewhat direction- and not speed-selective (Fig. 3, Supplementary Fig. 3) and the second-level units that are both direction- and speed-selective (Fig. 4) were derived from natural image movies, given the task of component-speed estimation. Thus, it is interesting to ask how the properties of these units compare with those of individual neurons in V1 and MT.

Simple and complex cells in V1 vary greatly in their selectivity to the sign of component-motion direction^2^,^18^,^44^. A study using antidromic stimulation showed that the subsets of V1 complex cells projecting to MT are component cells that are highly selective to the sign of motion direction^18^. Interestingly, this study showed that the distribution of the direction-selectivity index in this sub-population of V1 neurons is essentially the same as those of MT neurons, implying that MT neurons inherit their direction-selectivity from V1.

This result suggests a potential parallel between processing in V1 and the ideal observer for component speed estimation. The poor speed tuning of first-level units (Fig. 3a, Supplementary Fig. 4) is similar to that of the general population of V1 simple cells^14^. Also, the distribution of direction-selectivity for the first-level units (Supplementary Fig. 5a) is similar to that of the general population of simple and complex cells, and the distribution for the second-level speed-selective units (Supplementary Fig. 5b) is similar to that of V1 complex cells projecting to MT^18^. Therefore, speed-selectivity in MT (like direction-selectivity) may be inherited from a specialized subset of neurons in V1.

The broad distribution of direction-selectivity in the first-level units follows from an analysis of natural image movies based on maximizing accuracy in a speed estimation task. An analysis based on maximizing sparseness and completeness (efficient coding) yields linear space-time RFs^15^,^16^ that appear to be much less varied in their direction-selectivity (more direction selective on average) than those reported here. Thus, arbitrary subsets of these sparse-coding populations will be less optimal for speed estimation. Of course, accurate estimates of speed could, in principle, be obtained from any complete representation (just as the retina contains all available information for speed estimation). Therefore, it is possible that a small subset of these sparse populations are similar to the specific RFs identified here. If that turns out to be the case, then our results could also be described as identifying the best subset to pool for obtaining speed-selective neurons.

Many neurons in MT are speed tuned, have tuning curves that are approximately log-Gaussian over speed and have bandwidths that increase systematically with preferred speed^14^,^45^,^46^. These properties are consistent with the optimal second-level units (Fig. 4, although the second-level units have narrower bandwidths than neurons in MT). It has been argued that these properties are consistent with the psychophysical finding of Weber's law for speed discrimination^45^. Our analysis shows further that these properties and the resulting Weber's law (see Fig. 5) are consistent with optimal processing of natural signals, and hence are predicted from first principles.

### Alternative implementations of ideal computations

In the results section, we describe one way of implementing the ideal computations (see Fig. 4a). Although that implementation is relatively simple, the implementation of the L neurons is not biologically plausible, because strictly linear neurons do not exist in the visual system (for example, there are no spike rates below zero). However, in the Supplementary Material we describe a more biologically plausible implementation of the L neurons that is in the spirit of the classic model for obtaining complex cells; namely, by summing the responses of simple cells^2^.

Of course, there is no mathematical requirement that the likelihood of the joint RF responses be explicitly represented in a second population of L neurons, but available neurophysiology seems consistent with this story. Neurons in a number of well-documented brain areas (for example, areas V1 and MT) seem to represent variables explicitly that are represented only implicitly in earlier areas. As signals proceed through the visual system, neural states become more selective for properties of the environment, and more invariant to irrelevant features of the retinal images.

### General implications of the variability of natural signals

**Figure 9:**
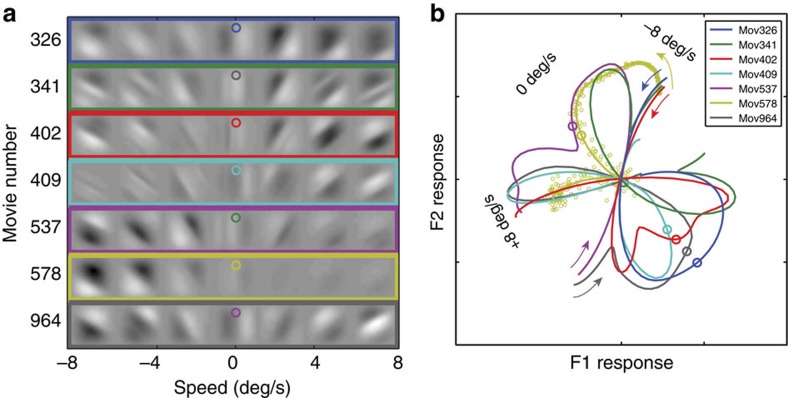
Natural stimulus variability. (**a**) Different movies in the test set. Rows show movies that all shared the same first frame (*c.f.*, Fig. 2a). Each row therefore represents the set of retinal image movies that would be produced by translating past the same point in a scene at different speeds. Columns show different speeds ranging between −8 and 8 deg/s. The variation across rows represents the fact that variable spatial-temporal frequency content and contrast is an irreducible form of stimulus variability in natural viewing, even when speed is the same. (**b**) Expected responses of the first two receptive fields (Fig. 3a) to movies sharing the same first frame across the full range of speeds (colour-code same as in **a**). Responses do not segregate by the starting frame of each movie (‘image identity') nearly as well as they segregate by speed (*c.f.*, Fig. 3c). The termination points of each curve indicate the responses to the fastest leftward (−8 deg/s, arrows) and rightward speeds (+8 deg/s) of each row in **a**. Speed changes with position along the curve. Large circles indicate responses at zero speed. Small circles show noisy joint responses to movie set 578. Noise magnitude in this response space reflects the combined effects of contrast normalization and noise added to equal the minimum equivalent noise in humans (see Results, equation (1), Fig. 4a). As contrast decreases (faster rightward speeds for movie 578), effective noise magnitude increases.

Natural signals are highly variable. Hence, to determine the optimal computations for natural signals or to evaluate how well an organism is processing natural signals, it is important to analyse large numbers of stimuli. Figure 9 helps make this point. The space-time plots within each coloured rectangle in Fig. 9a represent the set of retinal image movies that would be produced by translating past the same point in a scene at different speeds (*c.f.*, Fig. 2a). Each different coloured curve in Fig. 9b shows the corresponding joint response of the two RFs for each set of movies at different speeds.

There is great variation in the locus of joint responses produced by different natural stimuli, as a function of speed. It is obvious from these plots that attempts to determine the optimal computations from a small number of natural movies are likely to be frustrated, and conclusions about the optimal computations are likely to be mistaken (*c.f.*, Fig. 2c). Yet, much psychophysical and neurophysiological research focuses on characterizing responses to small sets of artificial stimuli where each stimulus is presented many hundreds of times.

Analysing a large representative set of naturalistic stimuli can complement more traditional experimental designs and provide a better picture of the processing required under natural conditions. In natural conditions, the exact same stimulus is rarely if ever seen twice. Thus, the variation and uncertainty in natural stimuli can be assets rather than hindrances for discovering the computations that optimize performance in critical sensory-perceptual tasks.

### Generality of findings and next steps

A number of different factors underlie the optimal RFs and estimation performance described here. First, the ideal observer analysis was applied to noisy photoreceptor responses that were computed using the optics of the human eye and the spatial density and temporal impulse–response function of human/primate photoreceptors reported in the literature. These factors have a substantial effect, because they filter out high spatial and temporal frequencies, but they must be modelled because they are essentially known factors that prevent information from reaching retinal and cortical circuits. Thus, to understand information processing in real systems, these factors are appropriate and essential to include.

Second, we included contrast gain control, a ubiquitous property of early visual processing^10^,^29^,^30^ (see Supplementary Note 1). This factor has little effect on the optimal RFs, because the normalization has no effect on the spatiotemporal shape of the signals.

Third, we analysed natural image movies that were created by texture-mapping image patches onto surfaces, and then translating the surfaces behind small apertures. Analysis was performed for both frontoparallel surfaces (rigid motion) and slanted surfaces (non-rigid motion). Differences in performance were slight although there were some differences in the optimal RFs (Supplementary Fig. 2). A limitation of our stimulus set was that it did not include the effects of occlusions and depth discontinuities (discontinuous motion). Spatially varying motion signals that vary across the retina may help define the spatiotemporal integration window (pooling area) that maximizes the accuracy of motion estimation^22^. A productive direction for future work would be to analyse co-registered range and camera images of real scenes captured during translation along known paths.

How important is the phase structure of natural image movies? We addressed this question by texture-mapping Gaussian noise with the amplitude spectrum of natural images (1/*f* noise) onto surfaces. Again, there are modest differences in performance and RFs. Training on drifting sinewaves and testing with natural movies result in more substantial differences. These results dovetail with previous results obtained for an ideal observer of disparity estimation in natural stereo-images^21^, and suggest that the ideal observer derived here for local speed estimates is relatively, but not completely, robust to modest changes in the stimulus properties.

The focus of this study was the estimation of component speed. A logical next step would be to consider how component-speed units (L neurons) for different orientations should be combined to obtain units that are tuned for specific motion vectors (speed and angular direction), like the pattern-motion cells found in area MT. One approach would be to sum the responses of appropriate subsets of component-speed units based on the ‘intersection of constraints' rule^12^,^13^,^23^. Another would be to take the responses of the component-speed units to natural image movies as input and repeat the analysis described here to obtain units optimal for motion-vector estimation.

## Conclusion

The present study used a Bayesian ideal observer analysis of natural signals to obtain principled hypotheses for perceptual processing in a specific natural task, and then tested the parameter-free quantitative predictions of those hypotheses in a behavioural experiment with stimuli derived directly from the natural signals. In a number of other recent studies^19^,^21^,^47^,^48^ ‘natural systems analysis' has provided new insights into the neural mechanisms and computational principles that underlie human performance in natural/naturalistic conditions. Evolution has pushed organisms towards the optimal solutions in critical sensory-perceptual tasks. It seems likely that this general approach will prove useful for other sensory-perceptual systems in a wide range of organisms.

## Methods

### Psychophysical methods

*Human observers*. Three observers participated in the experiments. All had normal or corrected-to-normal acuity. Two observers were authors; the third was naive to the purpose of the experiment. The human subjects committee at the University of Texas at Austin approved protocols for the psychophysical experiments. Informed consent was obtained.

*Stimuli*. Stimuli were presented on a Dell P992 19inch cathode ray tube monitor with 1,600 × 1,200 pixel resolution, and a refresh rate of 62.5 Hz. The display was linearized over 8 bits of grey level. The maximum luminance was 116.0 cd/m^2^; the mean background grey level was set to 58.0 cd/m^2^. The observer's head was stabilized with a chin- and forehead-rest, positioned 93 cm from the display monitor. Each stimulus movie subtended a visual angle of 1.0 deg (76 × 76 monitor pixels). Movie duration was 256 ms (16 frames at 62.5 Hz). All stimuli were windowed with a raised-cosine in space and a flattop-raised-cosine in time. The transition regions at the beginning and end of the time window each consisted of six frames (96 ms); the flattop of the window in time consisted of four frames (64 ms). Stimuli for the ideal observer were windowed identically. To prevent aliasing, stimuli were low pass filtered in space and in time before presentation (Gaussian with *σ*_*u*_=4 c.p.d., *σ*_*ω*_=31.25 Hz)^49^. No aliasing was visible. Sinewave stimuli were phase randomized on every trial, and were windowed and preprocessed identical to the natural stimuli.

All stimuli were set to have the same mean luminance as the background (58.0 cd m^−2^) and a r.m.s. contrast of 0.141 (equivalent to 0.20 Michelson contrast with sinewave stimuli). The r.m.s. contrast is given by


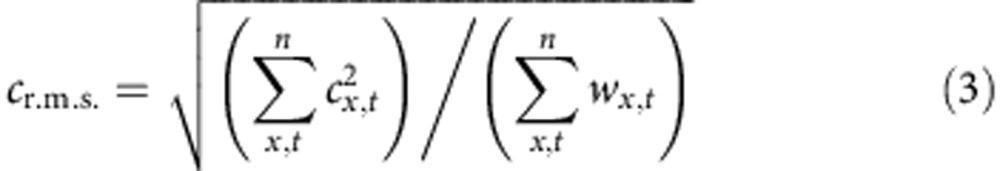


where *c*_*x*,*t*_ is the Weber contrast at each space-time pixel of the windowed stimulus, *w*_*x*,*t*_ is the space-time window and *n* is the dimensionality of the stimulus contrast vector. Stimuli were contrast-equalized because low contrast can have a strong effect on speed percepts^23^,^25^,^50^. By equalizing the on-screen contrast of the stimuli, we eliminated the possibility that contrast differences (between different movies having the same speed) are responsible for variation in human performance. Thus, our analysis predicts variation in speed estimation (that is, underestimation and overestimation) for movies having exactly the same speed and overall contrast. This variation is due to changes in the spatial-frequency content alone. Dependencies on contrast will be explored in future work.

*Procedure*. Data was collected using a two-interval forced choice procedure. The task was to select the interval with the movie having the faster speed via a key press; the key press also initiated the next trial. Feedback was given. A high tone indicated a correct response; a low tone indicated an incorrect response. On each trial, one ‘standard speed' and one ‘comparison speed' movie were presented in pseudo-random order. Movies always drifted in the same direction within a trial. Experimental sessions were blocked by absolute standard speed. An equal number of movies drifting to the left and right were presented in the same block to reduce the potential effects of adaptation. For example, in the same block, data was collected at standard speeds of −5.20 and +5.20 deg/s.

Psychometric functions were measured for each of 10 standard speeds (±5.20, ±4.16, ±3.12, ±2.08, ±1.04 deg/s) using the method of constant stimuli, seven comparison speeds per function. For each standard, each of the seven comparison speeds was presented 50 times. Each observer completed 3,500 trials (2 directions × 5 standard speeds × 7 comparison speeds × 50 trials).

The exact same naturalistic movie was never presented twice. Rather, movies were randomly sampled without replacement from the test set of 1,000 naturalistic movies at each speed (described in the main text). This sampling procedure was used to ensure that the set of stimuli used in the psychophysical experiment had approximately the same statistical variation as the stimuli that were used to train and test the ideal observer model. Specifically, for each standard speed, 350 ‘standard speed movies' were randomly selected. Similarly, for each of the seven comparison speeds corresponding to that standard, 50 ‘comparison speed movies' were randomly selected. Standard and comparison speed movies were then randomly paired together.

This feature of our experimental design represents a departure from methods used in classical psychophysical studies in which the same stimulus is presented many hundreds of times. Such studies have typically focused on the performance limits imposed by internal noise. In contrast, the aim of our study was to examine the performance limits imposed by variation and uncertainty in natural stimuli.

*Data analysis*. To compare performance, human and ideal observers were presented with the same randomly sampled, contrast-equalized stimuli. The ideal observer decision criterion was identical to the criterion the human observers were assumed to follow. Specifically,


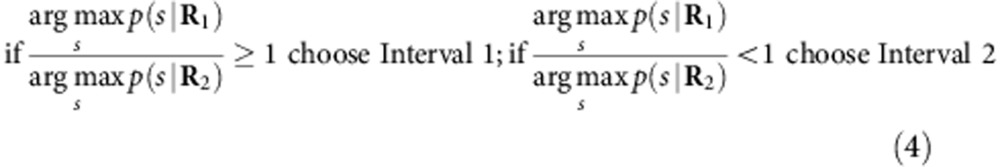


where **R**_1_ and **R**_2_ are the population responses of the space-time RFs (Fig. 3a) to the movies presented in the first and second intervals, respectively.

To obtain speed discrimination thresholds, the raw psychometric data was fit with a cumulative Gaussian function using maximum likelihood estimation. Threshold criterion was set to *d*′=1.36, the speed difference required to go from 50 to 75% on the psychometric function. Confidence intervals on the thresholds were calculated from 1,000 bootstrapped data sets. The psychometric data was similar for the two different directions of motion (left or right); data were collapsed across the direction of motion, and re-fit with the Gaussian. Thus, each symbol in Figs 5b and 6b,c is comprised of 100 measurements per absolute comparison speed, for a total of 700 measurements per psychometric function.

## Additional information

**How to cite this article:** Burge, J. & Geisler, W. S. Optimal speed estimation in natural image movies predicts human performance. *Nat. Commun.* 6:7900 doi: 10.1038/ncomms8900 (2015).

## Supplementary Material

Supplementary Information Supplementary Figures 1-5 and Supplementary Note 1-3 and Supplementary References
