# Supplementary material for: Optimal speed estimation in natural image movies predicts human performance
Source: Nat Commun. 2015 Aug 4;6:7900. doi: 10.1038/ncomms8900 (PMC4532855; doi:10.1038/ncomms8900)
Supplement: Supplementary Information — Supplementary Figures 1-5 and Supplementary Note 1-3 and Supplementary References [file ncomms8900-s1.pdf]

SUPPLEMENTARY FIGURES

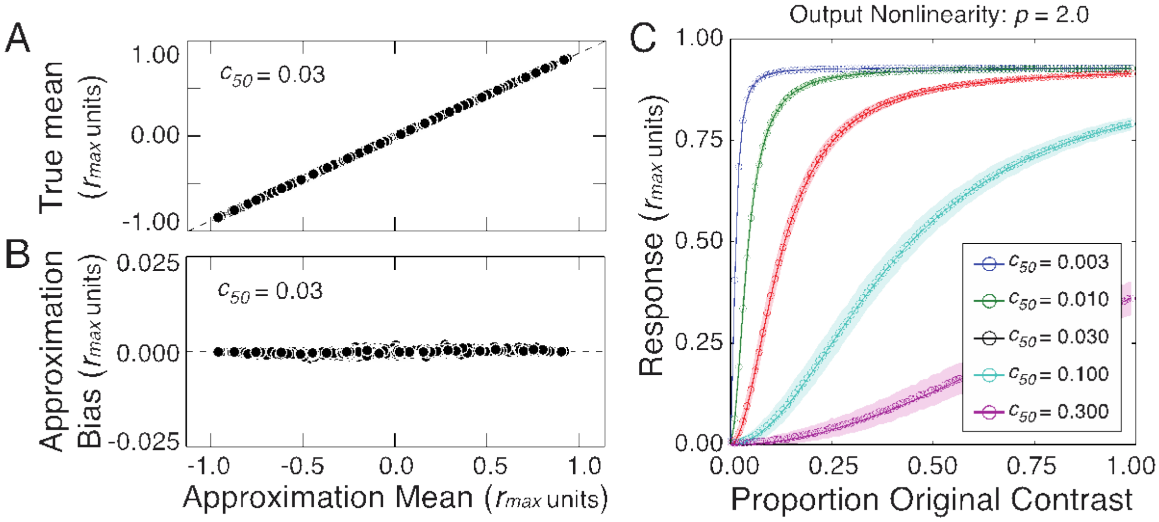

Supplementary Figure S1. Accuracy of contrast normalization approximation. **A** Accuracy of the approximation across all stimuli. Each circular symbol represents a different stimulus. The Approximation Mean (mean response given by Eq. S2) is plotted True Mean (sample mean response of Eq. S1; 10,000 samples). **B** The difference between the True Mean and the Approximation Mean. The average bias across stimuli is zero, meaning that the approximation is unbiased. Note the small scale of the y-axis. Additionally, root-mean-squared bias decreases asymptotically to zero with a power law, suggesting that residual bias is due to sampling error in the Monte Carlo simulation. Thus, approximation is accurate across the stimulus set. **C** Accuracy of the approximation for an individual stimulus. Response of a model simple cell to the stimulus in the training set that evokes the largest response from space-time receptive field $\mathbf{f}_1$, for original (100%) and artificially decreased (< %100) contrasts, for different values of c_{50}. The curves show the Approximation Mean (mean response given by Eq. S3 with output nonlinearity p = 2.0). The circular symbols show the True Mean (sample mean response of Eq. S1 with output nonlinearity p = 2.0; 10000 samples). Shaded areas show +/-1SD of noisy sample responses. The same result holds for all receptive fields and stimuli. The approximation is thus accurate for individual stimuli.

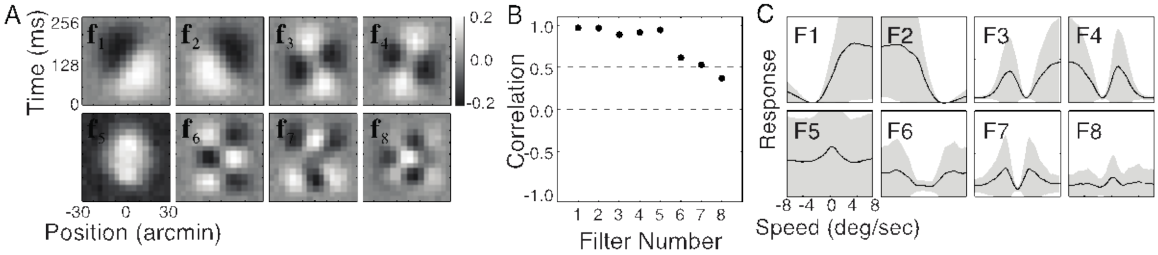

Supplementary Figure S2. The influence of training on movies with rigid motion only on optimal receptive fields for speed estimation. **A.** Optimal space-time receptive fields for speed estimation with only rigid retinal image motion. Just as in the main text, a training set was created by texture-mapping randomly sampled patches from photographs of natural scenes onto surfaces. The surfaces were then drifted behind an aperture. This training set included movies only of frontoparallel surfaces (rigid-motion only), whereas the set in the main text did not contained movies of surfaces slanted to varying degrees (non-rigid motion). The similarity between these receptive fields and those in the main text provides evidence that the results in the main text are largely robust. However, note that there exist some differences between these receptive fields and those presented in the main text. For example, receptive fields 6-8 appear more like discrete cosine transfer components than the receptive fields in cortex. **B.** Quantifying the similarity between individual receptive fields. Correlation between space-time receptive fields in main text (rigid motion), and the movies in A (non-rigid motion). The optimal space-time receptive fields are largely but not completely robust to whether the image set contains non-rigid and rigid vs rigid motion only.

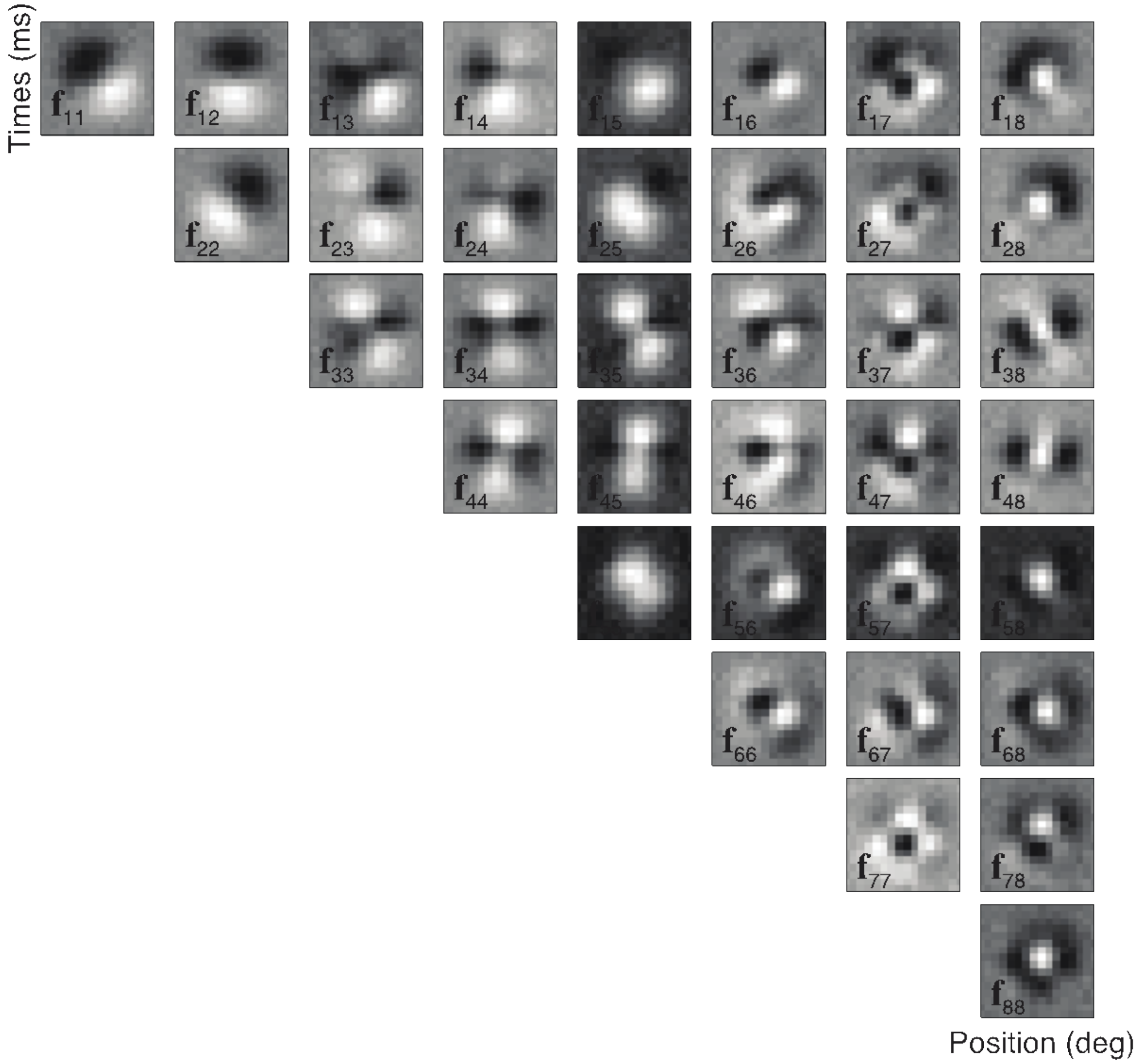

Supplementary Figure S3. Optimal space-time receptive fields and their pair-wise combinations. The original eight receptive fields are shown on the diagonal. The optimal computations could be implemented by appropriately weighting the squared responses of the receptive fields and their pair-wise combinations. This eclectic mix of receptive fields could be used in one of several possible implementations of the optimal computations (see Discussion, Supplement Note 3). Each receptive field response would get a different weight depending on the preferred speed of the likelihood neuron to which it contributes (equations S4,S5). Therefore, the variety of space-time receptive fields in cortex may play a functional role in speed estimation.

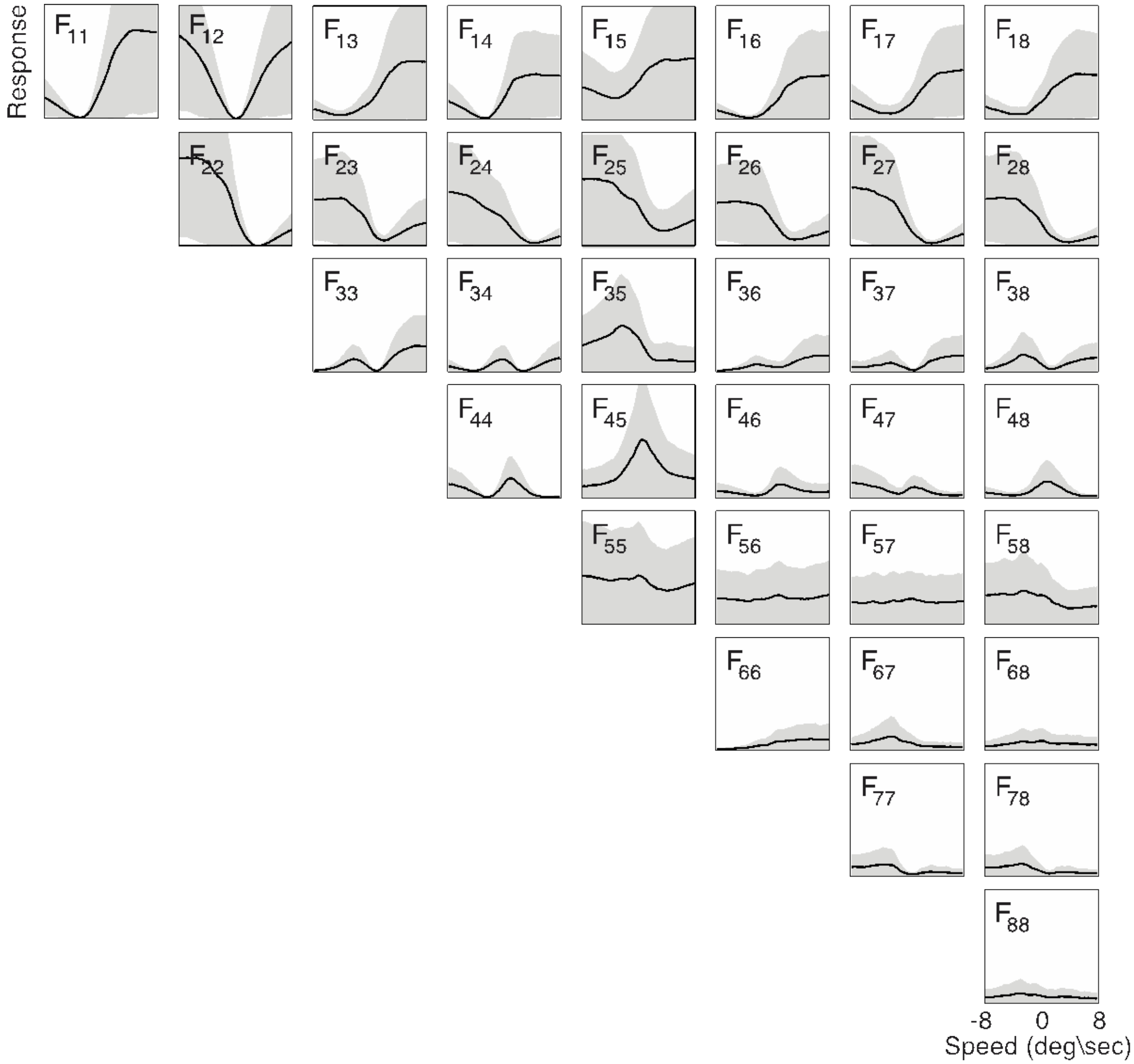

Supplementary Figure S4. The speed tuning curves of each optimal space-time receptive fields and their pair-wise combinations. The tuning curves of the original eight receptive fields are shown on the diagonal.

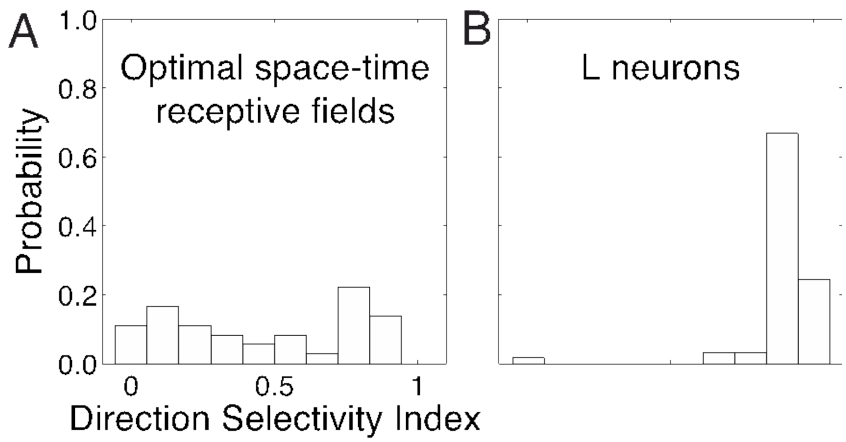

Supplementary Figure S5. Direction selectivity in first- and second-level units. **A**. Distribution of the direction selectivity indices for the first level units in the most biologically plausible implementation of the ideal observer for speed estimation (see Discussion, Supplementary Note 3, Supplementary Fig. S3, Supplementary Fig. S4). **B**. Distribution of the direction selective index for second level units (L neurons, see Fig. 4).

SUPPLEMENTARY NOTE 1

Contrast normalization

In the main text, we claim that the standard equation for contrast normalization in the literature provides a good approximation for the expected value of receptive field responses to encoded images that have been corrupted by noise. Here, we present Monte Carlo simulation results to support the claim. We examine the accuracy of the approximation for an individual image movie, and across the entire set of movies. Eq. (1) in the main text (repeated here) gives the response of a linear space-time receptive field to a noisy, contrast-normalized stimulus

$$R = \mathbf{f} \cdot \frac{(\mathbf{c}+\mathbf{n})}{\sqrt{\|\mathbf{c}+\mathbf{n}\|^2}} \qquad \text{(S1)}$$

where $\mathbf{n} \sim N(0,\sigma^2\mathbf{I})$ is i.i.d Gaussian noise with standard deviation σ. That is, equation S1 gives the response of a linear receptive field (with normalization).

Most simple cells incorporate two more nonlinearities: half-wave rectification (simple cells cannot produce negative responses) and a squaring nonlinearity. Together, these three nonlinearities (i.e. contrast normalization, rectification, & squaring) account for the characteristic shape of simple-cell contrast response functions. Incorporating these features into Eq. S1, yields

$$R = r_{\max} \left\lfloor \mathbf{f} \cdot \frac{(\mathbf{c}+\mathbf{n})}{\sqrt{\|\mathbf{c}+\mathbf{n}\|^2}} \right\rfloor^2 \qquad \text{(S2)}$$

where the half-bracket represents half-wave rectification. Thus, equation 2 gives the expected simple cell response to a noisy input image.

The standard contrast normalization model for V1 simple cell responses[1-3] is given by

$$r = r_{\max} \left\lfloor \mathbf{f} \cdot \frac{\mathbf{c}/\sqrt{n}}{\sqrt{\left(c_{RMS}^2 + c_{50}^2\right)}} \right\rfloor^2 \qquad \text{(S3)}$$

where c_{50} is the half-saturation constant, c_{RMS} is the root-mean-squared contrast of a stimulus (i.e., $c_{RMS} = \sqrt{\|\mathbf{c}\|^2/n}$), and n is the number of pixels in the contrast patch $\mathbf{c}$. Thus, Eq. S3 does not explicitly include the effects of input noise.

We asked whether the expected value of the model simple cell responses to noisy input images (Eq. S2) is a reasonable approximation to standard model responses to noiseless input images

(Eq. S3). We performed a Monte Carlo simulation to check whether $E\left[R\right]$ (see Eq. S2) is approximately equal to *r* (see Eq. S3). Without loss of generality we can set $r_{\max} = 1.0$. First, we examined the accuracy across the entire set of stimuli for the value of the standard deviation of the contrast noise used in our experiment ($\sigma = 0.03$). The results show that the approximation is unbiased across the stimulus set (Supplementary Fig. S1A). However, accuracy across the stimulus set does not guarantee that the approximation is accurate for individual stimuli. To examine whether the approximation holds for individual stimuli, we selected a stimulus from the training set that produced the largest response from space-time receptive field $\mathbf{f}_1$. Then we performed a series of Monte Carlo simulations (10000 samples each), for a range of σ values, as the contrast of the stimulus was manipulated. The solid curves in Supplementary Fig. S1C plot Eq. S3 as a function of stimulus contrast, and the circles plot the expected value of *R* (Eq. S2) from the Monte Carlo simulations. For all values of σ and all image movie contrast the approximation is very accurate. The same result holds for all receptive fields and stimuli. One implication of this result is that spatiotemporal noise in the retina could be contributing to the half-saturation constant c_{50} of cortical simple cells. If retinal noise were the only contributing factor, the half-saturation constant of cortical simple cells would equal the standard deviation of the noise in the photoreceptors ($c_{50} = \sigma$).

SUPPLEMENTARY NOTE 2

Weights for likelihood neurons

The weights for constructing the speed-tuned likelihood neurons (see Fig. 4a) are given by simple functions of the covariance matrix. Each covariance matrix $\mathbf{C}(s_k)$ represents the response covariance of the receptive fields for all movies having a particular speed s_k. We denote $\mathbf{C}(s_k)$ with $\mathbf{C}_k$ for notational simplicity. The weights on the squared and sum-squared filters (see Figs. 3, S4) are given by

$$\mathbf{w}_{ii,k} = -diag(\mathbf{C}_k^{-1}) + 0.5\mathbf{C}_k^{-1}\mathbf{1} \quad \text{(S4a)}$$

$$\mathbf{w}_{ij,k} = -0.5\mathbf{C}_{ij,k}^{-1}, \forall ij, j > i \quad \text{(S4b)}$$

where **I** is the identity matrix, **1** is the 'ones' vector, and *diag()* sets a matrix diagonal to a vector.

The response of the likelihood neuron with preferred speed s_k is then given by

$$R_k^L \propto \exp\left[\sum_{i=1}^{n} w_{ii,k} R_i^2 + \sum_{i=1}^{n-1}\sum_{j=i+1}^{n} w_{ij,k}\left(R_i + R_j\right)^2\right] \quad \text{(S5)}$$

The proportionality can be turned into an equality by adding a constant $const_k$ to the exponent having a value proportional to the log of the determinant of the covariance matrix $\mathbf{C}_k$.

A loose analogy can be made between the terms in equation S5 and the properties of neurons. The term in the brackets can be thought of as synaptic contributions to the polarization state of the likelihood neuron. The exponential function can be thought of as the non-linearity that converts voltage (which can be positive or negative) to spike rate (which is always positive).

SUPPLEMENTARY NOTE 3

Alternate implementations of ideal estimator

The implementation that is schematized in Fig. 4A starts with linear receptive fields corresponding to the AMA receptive fields, $\mathbf{f}_i$, and all their pairwise sums, $\mathbf{f}_{i+j}$ (Supplementary Figs. S3 & S4). The responses of these receptive fields, R_i and R_{i+j} are then squared, combined via an appropriately weighted sum, and passed through an accelerating nonlinearity to obtain the L neuron responses. An equivalent implementation is to start with the AMA receptive fields only, $\mathbf{f}_i$. The responses of these receptive fields and their pairwise sums— $\boldsymbol{R_i}$ and $\left(\boldsymbol{R_i} + \boldsymbol{R_j}\right)$, respectively—are then squared, combined in an appropriately weighted sum (as before), and passed through the same accelerating nonlinearity (as before) to obtain the L neuron responses. These two ways of implementing the ideal are compact and simple conceptually, but are not biologically plausible because linear neurons do not exist in cortex; neurons, for example, cannot respond with a negative spike rate.

A more biologically plausible implementation of the L neurons would be in the spirit of the classic model for obtaining complex cells; namely, by summing the responses of simple cells[4]. Simple cells are typically modeled as a linear filtering stage followed by half-wave rectification and a squaring output nonlinearity. The squared output of each optimal space-time receptive field (and their pairwise sums) could be obtained from a pair of matched on and off units mimicking standard V1 simple cells. The simple cell responses would then be summed with appropriate weights and passed through an accelerating nonlinearity (as before) to obtain the L neuron responses. In this implementation, the L neurons would be a specific type of complex cell optimized for speed estimation. (A special case of this implementation are so-called “energy” units, which are obtained by summing the responses of four simple cells corresponding to a pair of receptive fields in quadrature phase[5].) Complex cells for other tasks (e.g. disparity estimation) could be obtained analogously, but would require different receptive fields and weights[6]. All of the above ways of implementing the ideal estimator are mathematically equivalent. It remains uncertain how the brain might approximately implement such ideal calculations. However, the above arguments show that such calculations could be implemented with well-known neural operations.

SUPPLEMENTARY REFERENCES

1. Albrecht, D. G. & Geisler, W. S. Motion selectivity and the contrast-response function of simple cells in the visual cortex. *Vis. Neurosci.* **7,** 531–546 (1991).
2. Heeger, D. J. Normalization of cell responses in cat striate cortex. *Vis. Neurosci.* **9,** 181–197 (1992).
3. Heeger, D. J. Half-squaring in responses of cat striate cells. *Vis. Neurosci.* **9,** 427–443 (1992).
4. Hubel, D. H. & Wiesel, T. N. Receptive fields and functional architecture of monkey striate cortex. *J. Physiol. (Lond.)* **195,** 215–243 (1968).
5. Adelson, E. H. & Bergen, J. R. Spatiotemporal energy models for the perception of motion. *J Opt Soc Am A* **2,** 284–299 (1985).
6. Burge, J. & Geisler, W. S. Optimal disparity estimation in natural stereo images. *J Vis* **14,** (2014).
